# Fast Deoxynivalenol Determination in Cereals Using a White Light Reflectance Spectroscopy Immunosensor

**DOI:** 10.3390/bios10110154

**Published:** 2020-10-25

**Authors:** Vasileios Anastasiadis, Ioannis Raptis, Anastasios Economou, Sotirios E. Kakabakos, Panagiota S. Petrou

**Affiliations:** 1 Immunoassays-Immunosensors Lab, Institute of Nuclear & Radiological Sciences & Technology, Safety & Energy, National Center for Scientific Research “Demokritos”, 15310 Aghia Paraskevi, Greece; vanasta@chem.uoa.gr (V.A.); skakab@rrp.demokritos.gr (S.E.K.); 2 Analytical Chemistry Lab, Department of Chemistry, National and Kapodistrian University of Athens, Panepistimiopolis, 15771 Zografou, Greece; aeconomo@chem.uoa.gr; 3 Institute of Nanoscience & Nanotechnology, National Center for Scientific Research “Demokritos”, 15310 Aghia Paraskevi, Greece; i.raptis@inn.demokritos.gr; 4 ThetaMetrisis S.A., 12132 Athens, Greece

**Keywords:** deoxynivalenol, optical immunosensor, while light reflectance spectroscopy, cereal samples

## Abstract

Deoxynivalenol (DON) is a mycotoxin produced by certain *Fusarium* species and found in a high percentage of wheat and maize grains cultured worldwide. Although not so toxic as other mycotoxins, it exhibits both chronic and acute toxicity, and therefore methods for its fast and accurate on-site determination are highly desirable. In the current work, we employ an optical immunosensor based on White Light Reflectance Spectroscopy (WLRS) for the fast and sensitive immunochemical label-free determination of DON in wheat and maize samples. The assay is completed in 12 min and has a quantification limit of 2.5 ng/mL in buffer corresponding to 125 μg/kg in whole grain which is lower than the maximum allowable concentrations set by the regulatory authorities for grains intended for human consumption. Several extraction protocols have been compared, and the highest recovery (>90%) was achieved employing distilled water. In addition, identical calibration curves were received in buffer and wheat/maize extraction matrix providing the ability to analyze the grain samples using calibrators in buffer. Recoveries of DON from spiked wheat and maize grain samples ranged from 92.0(±4.0) to 105(±4.0)%. The analytical performance of the WLRS immunosensor, combined with the short analysis time and instrument portability, supports its potential for on-site determinations.

## 1. Introduction

**Figure 1:**
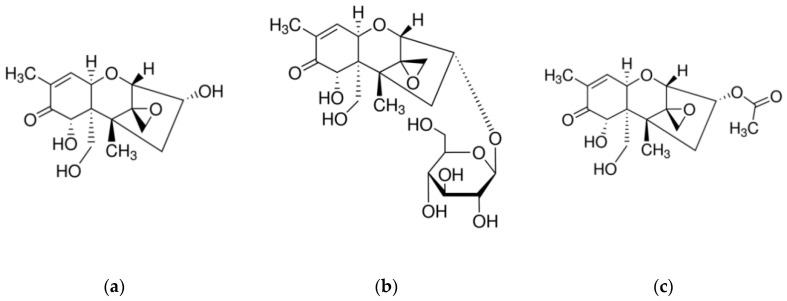
Chemical structures of (**a**) deoxynivalenol (DON); (**b**) DON-3-glucoside; and (**c**) 3-acetyl-DON.

*Fusarium* species are a large group of phytopathogenic fungi which infect cereal crops either in field during their growth or after crop during storage when the temperature and humidity conditions are suitable for their growth. Infection of crops by *fusarium* could have serious effects in animals and humans that will consume them mainly due to the presence of mycotoxins produced by the fungi. The mycotoxins produced by fusarium include: trichothecenes, zearalenone, and fumonisins [1]. Deoxynivalenol (DON), also known as vomitoxin due to its potent emetic properties, belongs to the trichothecene group and it is produced mainly by the species *Fusarium graminearum* and *Fusarium culmorum* [2]. It is the trichothecene most frequently detected in high amounts in cereals and as such it has been associated with health issues such as reduced food consumption, vomiting, weight loss, diarrhea, and immunological dysfunction, while it acts as an inhibitor of protein synthesis. DON is a polar organic compound, as it can be deduced by its chemical structure shown in Figure 1a, since it contains three hydroxyl groups which have been associated with its toxicity [3]. One of the most important physicochemical properties of DON is its stability at high temperatures which increases the risk of its presence in processed food derived from *fusarium*-infected cereals [4]. Numerous studies have documented that DON is very stable at temperatures ranging from 170 to 350 °C, with no reduction of its concentration after 30 min treatment at 170 °C [4]. However, the DON levels are reduced when cereal-based food was boiled, a finding that has been ascribed to the high solubility of DON in water, while no reduction of its concentration was observed after frying DON-contaminated food in oil [4]. DON is metabolized both by plants and animal and thus it can reach humans through the food chain in many different forms usually referred to as “masked mycotoxins” [5,6]. The major DON metabolite in plants is DON-3-glucoside (D3G; Figure 1b) which can be hydrolyzed in the gastrointestinal tract of animals, including humans, thus leading to exposure to increased DON levels [6]. DON derivatives with glutathione and sulfates have also been identified in several cereals. In mammals, DON received through food can follow one out of three metabolic pathways [6,7,8,9]. In the first, ingested DON is transformed by intestinal or ruminal microbes to de-epoxy-deoxynivalenol [7]. In the second, DON is conjugated to glucuronic acid forming deoxynivalenol-glucuronide, while in the third pathway, which seems to be a major detoxification route in mammals, DON undergoes sulfonation mainly at positions 3 and 15 [8,9]. In addition to these metabolites, DON is usually produced along with its acetylated-derivatives, 3-acetyl-DON (3AcDON; Figure 1c) and 15-acetyl-DON [10,11]. Although not enough toxicity data are currently available regarding all DON metabolites and derivatives, their existence in food indicates that DON might be a more severe problem than is anticipated based on its toxicity only. The problem is intensified by the fact that a high percentage of cereals used for human consumption have been found contaminated with DON [12].

To protect consumers from exposure to high DON levels, regulatory authorities worldwide have set maximum levels for both unprocessed and processed cereals [13,14]. For example, EU has set as maximum levels for DON 1250 μg/kg in unprocessed cereals other than durum wheat, oats and maize, 1750 μg/kg in unprocessed durum, wheat, oats and maize, 750 μg/kg in cereals intended for direct human consumption, cereal flour, bran and germ, as well as for pasta, 500 μg/kg for bread, pastries, cereal snacks and breakfast cereals, and 200 μg/kg for cereal-based foods and baby foods for infants and young children [13]. At the same time, FDA has established advisory levels of 1000 μg/kg for finished wheat products (flour, bran, and germ), 10 mg/kg for grains and grain by-products, and 30 mg/kg for distillers and brewers grains, while the levels for animal feed range from 5 to 10 mg/kg [14].

As a response to the need for testing cereals and cereal-based products against DON, a variety of analytical procedures have been reported in the literature [15,16,17]. The methods include high performance liquid chromatography coupled to mass spectrometry (LS-MS/MS) [18,19] or fluorescence detectors after appropriate derivatization [20], which are also the reference methods for DON determination in food and feed. These methods are characterized by high detection sensitivity and ability for multiplexed mycotoxins determination in a single run [16,17]. Nonetheless, all these methods are laboratory-bound with high needs in both labor and time. Immunochemical methods, on the other hand, are considered more rapid and suitable for on-site determinations [16,17,21], especially in the form of immunochromatographic strips which, however, provide only semi-quantitative results [22,23,24,25,26,27]. Quantitative results could be acquired in relatively short time with standard microplate enzyme-immunoassays [25,28,29,30,31,32,33,34,35], which are less labor demanding compared to chromatographic techniques but still more suitable for laboratory rather than for field use. The potential for quantitative determinations combined with short analysis time and on-site application through the implementation of portable instruments is offered only by biosensors [16,17,21]. To this end, several types of biosensors have been employed for mycotoxin detection in food samples. Amongst those, the optical ones, and especially the label-free optical sensors, demonstrate several advantages in terms of sensitivity, short analysis time, potential for multiplexed analysis and implementation with portable instruments. For DON determination, label-free immunosensors based on surface plasmon resonance (SPR) [35,36,37], imaging SPR (iSPR) [38,39], biolayer interferometry (BLI) [35,40,41], and integrated onto silicon chips planar Mach–Zehnder interferometers (MZI) [42] have been reported in the literature. With a few exceptions [38,42], these sensors rely on bench top instruments not easily adaptable to on-site determinations.

**Figure 2:**
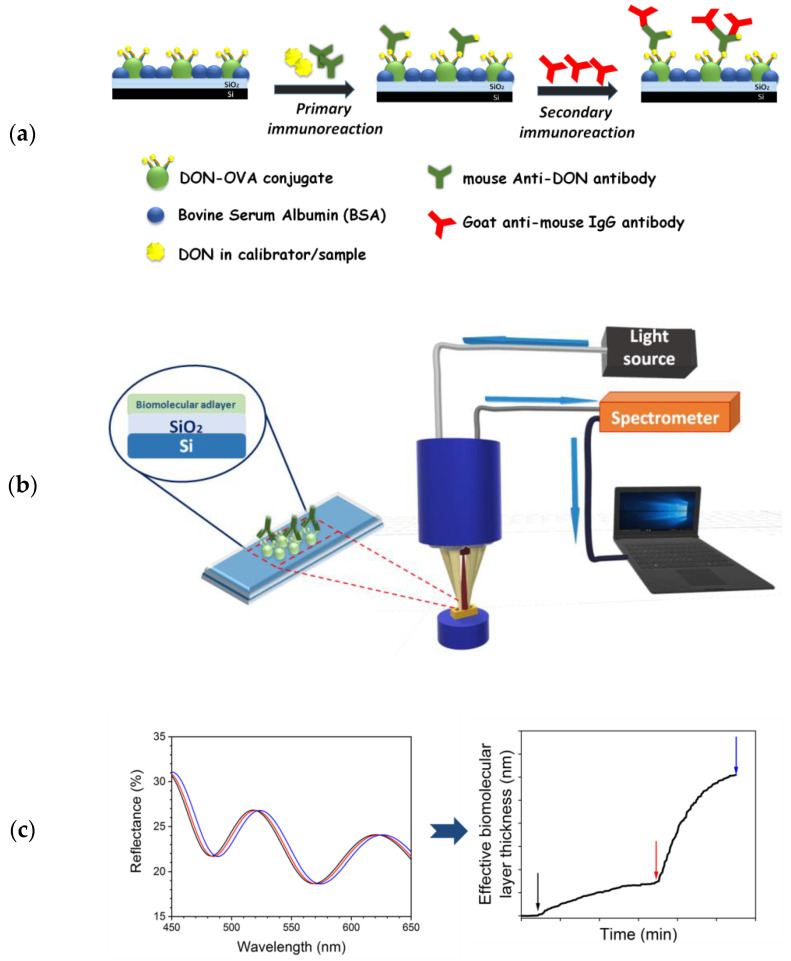
(**a**) Schematic of the immunoassay format and steps. (**b**) Schematic of the instrumentation employed for DON determination. (**c**) The transformation of spectral shifts due to primary (red line in the left graph) and the secondary immunoreaction (blue line in the left graph) with respect to baseline (black line in the left graph) to “effective biomolecular layer thickness” (the black, red and blue arrows in the right graphs correspond to baseline, end of primary immunoreaction, and end of secondary immunoreaction, respectively).

In the current work, we employ a White Light Reflectance Spectroscopy (WLRS) sensing platform to develop a fast method for the immunochemical determination of DON in wheat and maize whole grain samples. The analytical performance of the WLRS sensing platform has been demonstrated in several applications related to human disease diagnostics [43,44] and food safety [45,46]. In addition, the low cost of consumables and required instrumentation, as well as the ability to combine all optical components and liquid handling modules in a small size instrument, makes the WLRS platform appropriate for field applications. For the determination of DON with the WLRS platform, a competitive immunoassay format accomplished in two steps was followed. First, the competitive binding of anti-DON mouse monoclonal antibody between the DON molecules in the sample and those immobilized onto the chip surface in the form of protein conjugate took place, and in a second step, a secondary antibody was introduced to achieve signal enhancement. A schematic of the assay format is provided in Figure 2a. Several methods for DON extraction from spiked grain samples were explored to determine the one providing the highest recovery values but also in order to establish an extraction method that would be simple enough to be applied for on-site determinations. The developed WLRS sensor reached quantification limits lower than the maximum allowable concentrations set by the different regulatory authorities for grains intended for human consumption.

## 2. Materials and Methods

### 2.1. Materials

Mouse monoclonal antibody against DON (anti-DON mAb), DON conjugate with ovalbumin (DON-OVA), and DON for calibrators’ preparation were purchased from Aokin AG (Berlin, Germany). Highly pure methanol, ethanol and acetonitrile (CHROMASOLV^®^ for HPLC, ≥99.9%), DON-3-glucoside, and 3-acetyl-DON were obtained from Sigma-Aldrich (Darmstadt, Germany). Bovine serum albumin (BSA) and (3-aminopropyl)triethoxysilane (APTES) were purchased from Acros Organics (Geel, Belgium). Goat anti-mouse IgG antibody (affinity purified) was from Merck Millipore (Darmstadt, Germany). The water used throughout the study was distilled. The QuEChERS extraction kits (OMK1-MP, EKK1-MP and ACK1-MP) were from CHROMAtific UG (Heidenrod, Germany).

### 2.2. Instrumentation

The WLRS measurement set-up used in the current study consists of: (a) the optical module for the illumination of the biochip and the recording of the reflectance spectrum, (b) the docking station for the placement of the biochip, and (c) the external fluidic circuit for the supply of the reagents (Figure 2b). The optical module consists also of three elements: a halogen tungsten light source (ThetaMetrisis S.A.), a miniaturized USB-controlled spectrometer (Ocean Insight Inc.) operating in the visible range, and a proprietary designed reflection probe (ThetaMetrisis S.A.). The biochip is a 5 mm × 15 mm Si chip with a 1-micron-thick thermal SiO_2_ overlayer (see Figure 2b) and it is covered by a custom designed microfluidic cell (Jobst Technologies GmbH; Freiburg, Germany) that provides the fluidic connections between the reagents’ containers and the micropump. The reflected spectrum is recorded continuously throughout the assay from the spectrometer applying an integration time of 60 ms, average of 15 spectra, and record of 1 spectrum per second. The spectra are processed in real-time by the dedicated software developed by ThetaMetrisis S.A., and the bioreactions taking place on the chip surface are expressed as “effective adlayer thickness” in nm (Figure 2c) [43,44]. The latter is the analytical signal provided by the sensor.

### 2.3. Preparation of Calibrators and Samples

A DON stock solution with concentration of 1 mg/mL was prepared in an 80:20 (*v*/*v*) methanol/water mixture and stored in aliquots at −20 °C. Calibrators were prepared in 50 mM Tris-HCl, pH 7.8, 9 g/L NaCl, 5 g/L BSA, 0.5 g/L NaN_3_ (assay buffer) by addition of appropriate amounts from the stock solution, and kept aliquoted for up to 2 months at 4 °C. The whole grain cereal samples (maize and wheat) were kindly donated by ATP r&d S.r.l. (36043 Camisano, Vicentino, Italy) after analysis with an LC-MS/MS method (LOQ 50 μg/kg) and were found not to contain detectable concentrations of DON. The whole grain samples were finely ground using a household electric coffee bean grinder. For the recovery experiments, 2 g of ground sample was mixed with 0.5 mL of DON solutions of different concentrations in methanol and left overnight to dry in a fume hood. Then, the ground samples were mixed with 10 mL of water in 50-mL centrifuge tubes and extraction was performed under shaking for 30 s. In the tubes were then added the contents of the QuEChERS extraction kit and the mixture was shaken for 1 min. Two hundred microliters from the liquid were transferred to Eppendorf tubes and centrifuged at 2000× *g* for 5 min using a Fast Gene^®^ Mini Centrifuge (Nippon Genetics Europe, Germany). Alternatively, the ground grain samples were mixed with 10 mL of distilled water and extracted under continuous shaking for 1h, prior to centrifugation. The supernatants collected with both extraction procedures were diluted prior to the assay 10 times with assay buffer. Percent recovery (%R) was calculated by applying the following equation:

(1) $$\%R\;=\;(\frac{DON\;amount\;determined\;in\;spiked\;sample-DON\;amount\;in\;sample\;prior\;to\;spiking}{DON\;amount\;added\;in\;the\;sample})\;\times \;100$$

### 2.4. Chip Bio Functionalization and Assay Protocol

The Si chips were cleaned through successive sonication for 10 min in baths of acetone and 2-propanol. After drying with N_2_, the chips were immersed in Piranha solution (1:1 H_2_SO_4_/30% (*v*/*v*) H_2_O_2_ mixture) for 20 min and then washed thoroughly with distilled water prior to immersion in a 2% (*v*/*v*) APTES solution in distilled water. The chips were incubated for 20 min, washed with distilled water and then cured for another 20 min at 120 °C. The APTES-modified chips were left at room temperature for at least 48 h prior to spotting the DON-OVA solution, while they could be used for up to 4 weeks after their preparation. The APTES-modified chips were spotted with a 200 μg/mL DON-OVA solution in 50 mM carbonate buffer, pH 9.2, using the BioOdyssey Calligrapher Mini Arrayer (Bio-Rad Laboratories, Inc.). In order to cover a 3 mm × 3 mm area at the center of the chip, multiple overlapping spots with a mean diameter of 400 μm were deposited. The spotted chips were incubated overnight in a high humidity chamber (75% humidity) prior to immersion in 0.1 M NaHCO_3_ solution, pH 8.5, containing 10 g/L BSA, for 1 h at room temperature. The chips were then washed with distilled water, dried under a N_2_ flow, and assembled with the microfluidic cell. For the assay, chips were first equilibrated by running a 50 mM Tris-HCl, pH 7.8, 9 g/L NaCl, 5 g/L BSA, 0.5 g/L NaN_3_ (assay buffer), then a 1:1 volume mixture of calibrators or samples with a 3 μg/mL anti-DON mAb in assay buffer was run for 7 min, followed by a 10 μg/mL anti-mouse IgG solution in assay buffer for 5 min. All solutions were run at a constant flow rate of 30 μL/min. The chip was regenerated by running for 3 min a 0.1 M HCl solution, and was equilibrated with assay buffer prior to the next run.

## 3. Results

### 3.1. Assay Optimization

**Figure 3:**
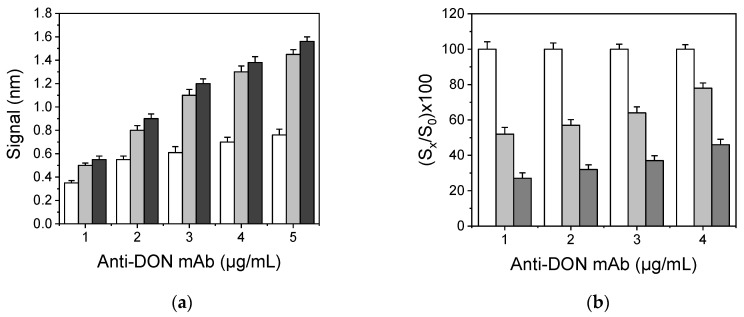
(**a**) Effect of mouse monoclonal antibody against DON (anti-DON mAb) concentration on the zero calibrator signal received from chips coated with 100 (white columns), 200 (light grey columns), or 400 μg/mL DON conjugate with ovalbumin (DON-OVA) (dark grey columns). (**b**) Effect of anti-DON mAb concentration on the percent signal corresponding to calibrators containing 10 ng/mL (light grey columns) and 100 ng/mL DON (dark grey columns) with respect to zero calibrator signal (white columns) obtained from a chip coated with 200 μg/mL of DON-OVA conjugate. In all cases, the duration of both primary and secondary immunoreaction was 10 min. Each point is the mean value of 3 measurements ± SD.

The DON assay developed on the WLRS biosensing platform is a competitive immunoassay performed in two steps. In the first step (primary immunoreaction), DON present in calibrators or samples competes with the DON immobilized onto the chip surface (in the form of DON-OVA conjugate) for coupling to the anti-DON mAb binding sites. Then, a secondary antibody (goat anti-mouse IgG antibody) is introduced which binds to the anti-DON mAb molecules bound to the chip surface in the first step, resulting in an enhancement of the signal received by the primary immunoreaction since more than one molecule of secondary antibody could react with a single primary antibody molecule (Figure 2a). In this assay format, the highest signal is obtained in absence of DON (zero calibrator), whereas by increasing the DON concentration in calibrators or samples the signal is reduced. The assay sensitivity, i.e., the ability to discriminate between DON concentrations as well as the lowest detectable concentration, depends almost exclusively on the binding affinity of the primary antibody and it is experimentally optimized for a given antibody through selection of appropriate concentrations of DON-OVA conjugate used for coating of the chips and anti-DON mAb as well as on the duration of the primary immunoreaction. Therefore, the first parameters optimized were the concentrations of DON-OVA conjugate and anti-DON mAb based on both the absolute maximum signal, i.e., the signal corresponding to zero calibrator, and the percent signal drop with respect to zero calibrator signal obtained for certain DON calibrators. As shown in Figure 3a, where the zero calibrator signals from chips coated with DON-OVA conjugate concentrations ranging from 100 to 400 μg/mL and anti-DON mAb concentrations from 1 to 4 μg/mL are presented, increase in DON-OVA conjugate concentration from 100 to 200 μg/mL resulted in considerable signal increase especially for the higher anti-DON mAb concentrations tested. On the other hand, further increase in DON-OVA conjugate concentration to 400 μg/mL caused a marginal increase to zero calibrator signal (approx. 10%), and therefore the concentration of 200 μg/mL was selected for spotting of the chips. Then, the anti-DON mAb concentration was selected based on the % signal drop determined for DON calibrators containing 10 and 100 ng/mL with respect to zero calibrator signals. As can be deduced by the data depicted in Figure 3b, the percent signal drops determined for the two calibrators were higher for anti-DON mAb concentration lower than or equal to 2 μg/mL. Nonetheless, the absolute signals obtained for those anti-DON mAb concentrations were rather low, limiting the assay’s dynamic range. Therefore, and in order to combine the higher possible zero calibrator signal with the higher signal drop in presence of DON, a 3 μg/mL anti-DON mAb concentration was selected, since further increase in the anti-DON mAb concentration reduced the percent signal drop observed for the calibrators negatively affecting the assay sensitivity.

**Figure 4:**
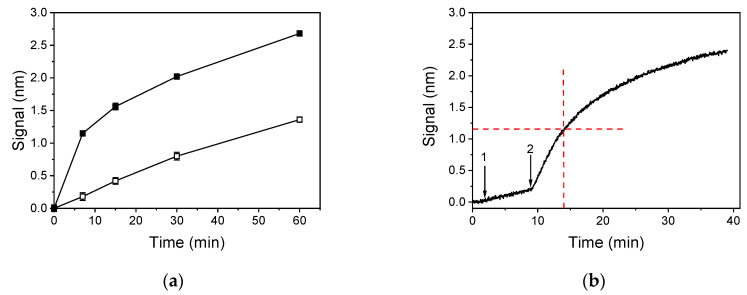
(**a**) Effect of primary immunoreaction duration on the zero calibrator signal without (open squares) or with an additional 5-min reaction with an anti-mouse IgG antibody (closed squares). Each point is the mean value of 3 measurements ± SD. (**b**) Real-time response corresponding to zero DON calibrator when running over a chip coated with a 200 μg/mL DON-OVA solution: assay buffer (start to arrow 1; 2 min), 1:1 (*v*/*v*) calibrator/anti-DON mAb mixture (arrow 1 to 2; 7 min), and anti-mouse IgG antibody (arrow 2 to 3; 30 min). The dashed red lines indicate the signal received for running the anti-mouse IgG antibody for 5 min.

For reasons related to the ease of the proposed assay application to routine analysis, it would be preferable that the determination is completed in one step, i.e., by monitoring the primary immunoreaction of the anti-DON mAb between DON in the sample and that immobilized onto the chip surface. However, as depicted in Figure 4a, the primary immunoreaction duration should be extended to 60 min in order to receive a signal equal to or higher than 1 nm. On the other hand, when a secondary immunoreaction step was introduced, i.e., further reaction with an anti-mouse IgG antibody, the same signal level was obtained for the shortest primary immunoreaction duration tested (i.e., 7 min) after only 5-min reaction with the secondary antibody. Despite the fact that, as shown in Figure 4b, the reaction with secondary antibody does not reach maximum plateau values even after 30 min, almost 50% of the signal obtained in 30 min is acquired within the 5 first min. Thus, for the final assay protocol of WLRS DON determination, a 7-min primary immunoreaction followed by 5-min reaction with the anti-rabbit IgG antibody solution was selected.

### 3.2. Analytical Characteristics of DON Immunosensor

**Figure 5:**
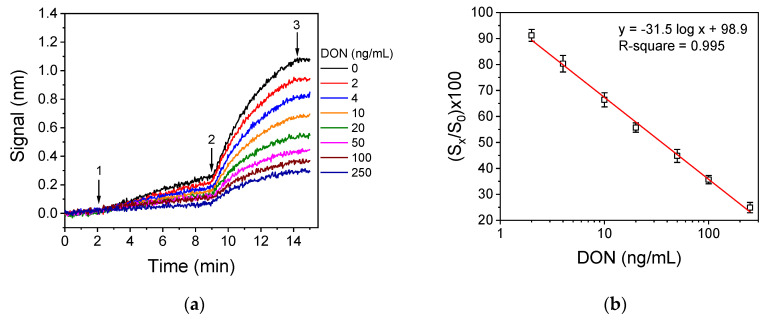
(**a**) Real-time responses obtained for DON calibrators with concentration 0–250 ng/mL. The solutions which were flowed over the chips were as follows: assay buffer (start to arrow 1; 2 min), 1:1 (*v*/*v*) calibrator/anti-DON mAb mixture (arrow 1 to 2; 7 min), anti-mouse IgG antibody (arrow 2 to 3; 5 min), and assay buffer (arrow 3 to end; 1 min). (**b**) Typical linearized DON calibration curve (Equation (2)) obtained with calibrators prepared in buffer. The linear regression equation and the coefficient of correlation values are provided in the figure. Each point is the mean value of 3 measurements ± SD.

The real-time sensor responses obtained for DON calibrators prepared in buffer with concentrations ranging from 0 to 250 ng/mL, following the final assay protocol, are provided in Figure 5a. As shown, it was not possible to discriminate the responses corresponding to the different calibrators just by monitoring the primary immunoreaction. Nonetheless, the responses accounted to different calibrators are quite distinct after the secondary immunoreactions, justifying the need for a signal enhancement step. Taking into account the end point signal values corresponding to calibrators, the curve presented in Figure 5b was linearized in a log/linear scale. The linear regression equation was:

(S_x_/S_0_)% = 98.9(±1.6) − 31.5(±0.9) × log(DON concentration) (2)

where S_x_ were the signals obtained for the calibrators containing DON and S_0_ the signal of the zero calibrator.

Then, the limits of detection and quantification were determined as the concentrations corresponding to (S_x_/S_0_)% values equal to 100 minus 3 standard deviations and 100 minus 6 standards deviations of the mean value of 20 measurements of zero calibrator. Thus, the assay’s detection and quantification limit were found to be 1.25 and 2.5 ng/mL, respectively. The assay linear working range extended up to 250 ng/mL.

### 3.3. Evaluation of Assay Specificity

**Figure 6:**
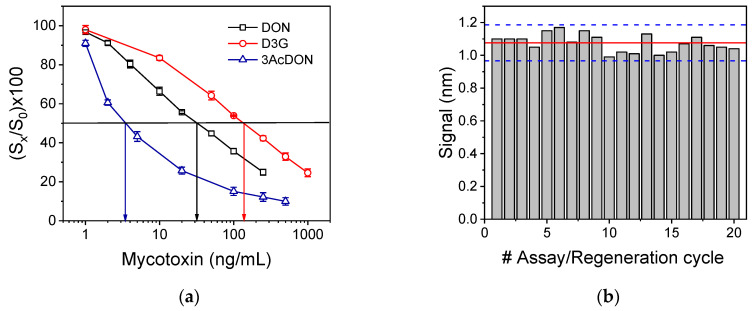
(**a**) Calibration curves obtained for DON (open squares), 3-DON-glycoside (open circles), and 3-acetyl-DON (open triangles) using chips coated with DON-OVA. The horizontal to *x*-axis black line corresponds to (S_x_/S_0_) value of 50%, whereas the vertical to *x*-axis blue, black, and red lines indicate the mycotoxin concentration that corresponds to 50% (S_x_/S_0_) for each one of the respective calibration curves. (**b**) Zero calibrator signals obtained from a single chip upon repetitive assay/regeneration cycles. The red solid line indicates the mean value of the 20 measurements and the blue dashed lines the ± 2SD limits. Each point is the mean value of 3 measurements ± SD.

Prior to application of the immunosensor developed for analysis of grain samples, the cross reactivity of the antibody implemented in the assay towards the major plant DON metabolite, 3-DON-glycoside (D3G), and a DON-derivative also produced by *Fusarium* species, 3-acetyl-DON (3AcDON), was determined. The calibration curves obtained with D3G and 3AcDON calibrators in chips coated with DON-OVA as well as the respective DON calibration curve are presented in Figure 6a. The percent cross-reactivity (%CR) was determined by applying the equation:

(3) $$\%\mathrm{CR}\;=\;(\frac{DON\;concentration\;corresponding\;to\;50\%\;signal\;drop}{Cross-reactant\;concentration\;corresponding\;to\;50\%\;signal\;drop})\;\times \;100$$

The concentrations of DON, 3-DON-glycoside, and 3-acetyl-DON that corresponded to 50% signal drop were 31.6, 137, and 3.4 ng/mL, respectively. Thus, the cross-reactivity against 3-DON-glycoside was found to be 23.0% and against 3-acetyl-DON 929%. The cross-reactivity with 3-DON-glycoside is not expected to interfere with DON determination in grain samples since as it has been reported in the literature the molar ratios of 3-DON-glycoside to DON range from 0.05 to 0.2 [47]. On the other hand, the extremely high cross-reactivity of anti-DON antibodies against with 3-acetyl-DON it has also been reported by other investigators and attributed to the fact that acetylated DON derivatives have been used as immunogens to raise these antibodies [28,48]. Despite this, the acetylated forms of DON (both 3-acetyl-DON and 15-acetyl-DON) are less frequently found in cereal samples compared to DON and when detected, their concentration is usually tens or even hundreds of times lower than that of DON [10].

### 3.4. Chip Stability and Regeneration Potential

The regeneration of the DON-OVA modified chip was further investigated in order to explore whether the chip can be reused without loss of its bioanalytical characteristics thus reducing the analysis cost. For this purpose, we tested several solutions that have been proposed in literature or employed in our previous works for regeneration of the chip [42,43,44,45,46]. In particular, the solutions evaluated for regeneration were: glycine-HCl buffer, pH, 2.5; 0.05 and 0.1 M HCl; 0.05 and 0.1 M NaOH; 0.5% (*w*/*v*) SDS, pH 1.3. These solutions should disrupt the antigen-antibody bonds ideally without affecting the immobilized molecules. Therefore, in addition to the ability of the regeneration solution to remove the bound anti-DON and secondary antibodies, their effect onto the immobilized DON-OVA conjugate was determined. The regeneration efficiency of each one of the solutions was evaluated by running each one of them over the chip for 5 min after the completion of an immunoassay cycle and, after equilibration with assay buffer, passing the secondary antibody solution in order to detect primary antibodies not removed during regeneration. Following this procedure, it was found that 0.1 M HCl solution provided almost complete removal of bound-to-surface antibodies (the signal obtained from the secondary antibody after regeneration was lower than 2% of the zero calibrator signal, and was constant for repetitive assay/regeneration cycles) and was therefore selected. The minimum duration of the regeneration step was also determined to be 3 min. In addition, following the selected regeneration procedure, repetitive cycles of assay/regeneration were performed to determine the effect on the immobilized DON-OVA conjugates and the possibility to reuse a single chip. As shown in Figure 6b, a single chip could be used up to 20 times after regeneration since the zero calibrator signals obtained were within the mean value ± 2SD limits.

### 3.5. Optimization of Grain Extraction Protocol

DON is more water soluble than other mycotoxins, such as for example aflatoxins, and therefore distilled water or mixtures of organic solvents with water are more efficient in extracting DON from whole grain samples rather than pure organic solvents. Thus, the extraction protocols tested included pure distilled water and water mixture with methanol (MeOH) and acetonitrile (AcN) at ratios ranging from 20:80 to 80:20 organic solvent/water. All protocols were tested by spiking a maize sample that did not contained detectable concentration of DON when analyzed with an LC-MS/MS method. As it can be concluded from the results presented in Table 1, extraction with distilled water provided percent recovery values for the three spiking levels tested. Regarding the recoveries achieved with mixtures of organic solvents and water, in case of methanol they were increased as the water content in the mixture was increased and recoveries similar to that obtained for water were achieved using mixtures 20:80 methanol/water. On the other hand, use of acetonitrile/water mixtures of 80:20 and 20:80 provided statistically similar results. Thus, the inclusion of organic solvent in the extraction medium is not considered necessary. A drawback of this extraction method is that to achieve high recovery, shaking of the sample with the water for at least 1 h was required. To reduce the extraction time the use of QuEChERS extraction kit, which is the most commonly employed food sample preparation technique, was investigated. In particular, three different kits were evaluated corresponding to three different sample preparation methods (OKM1: ORIGINAL; AKM1: AOAC 2007; EKM1: EN 15662). As shown in Table 1, there were not statistically significant differences in the recovery values determined when applying the three kits. However, the implementation of the kit reduced the time required for sample preparation to less than 10 min as anticipated with the more than 1 h procedure without the kit. Similar results regarding extraction efficiency have been received with spiked wheat samples.

**Figure 7:**
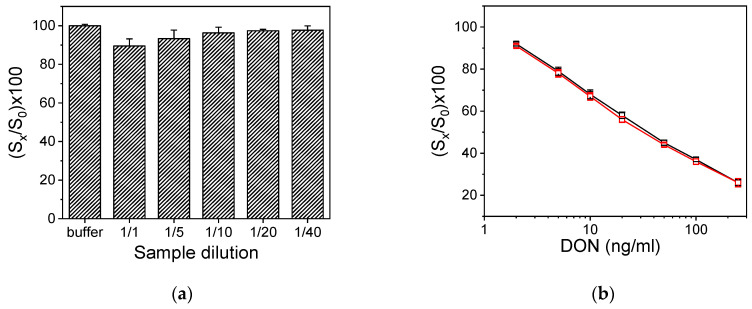
(**a**) Effect of maize water extract dilution with assay buffer to zero calibrator signal values with respect to zero calibrator value in buffer. (**b**) DON calibration curves received with calibrators prepared in assay buffer (black squares) or in maize water extract diluted ten times with assay buffer (red squares). Each point is the mean value of 3 measurements ± SD.

In the recovery experiments, the water extract was diluted 10 times with assay buffer prior to assay since it was found that this dilution affected neither the absolute zero calibrator signal nor the calibration curve. Nonetheless, higher as well as lower dilution factors were tested since the ability to use less diluted samples would lead to lower detectable DON concentrations in the grain samples. As shown in Figure 7a, dilution equal to or higher than 10 times led to zero calibrator signals that were statistically the same as the signal received in buffer. The zero calibrator signal was marginally affected when a dilution factor of 5 was applied; nonetheless this dilution could be applied if a lower detection is desirable. For the dilution selected, the effect in calibration curve was also examined. It was found that for the calibration curve obtained with calibrators prepared in maize or wheat water extract, ten-times dilution with assay buffer was identical to that obtained with calibrators prepared in buffer (Figure 7b), thus there is no need to use matrix-matched calibrators for DON determination with the proposed sensor.

Using the spiked maize and wheat samples and applying the sample preparation procedure selected, the intra-assay and inter-assay coefficients of variation (CVs) were calculated. In particular, the intra-assay CV was calculated from four repetitive measurements of sample within the same day, while the inter-assay CVs from four measurements were carried out in four different days in a period of 20 days and their values ranged from 2.1 to 6.3% and 3.5 to 8.2%, respectively.

## 4. Discussion and Conclusions

The DON assay developed in the WLRS platform was realized in 12 min or 17 min if regeneration and equilibration with buffer prior to and after the assay for 1 min were taken into account. The assay limit of detection (LOD) was 62.5 μg/kg for both wheat and maize samples, while the linear response range extended up to 12.5 mg/kg. The WLRS assay developed in the present work is compared in Table 2 in terms of LOD and assay duration with the most recent optical biosensors reported in the literature for DON determination in cereals or cereal-based products. The list includes two SPR sensors [36,37], both employing a bench top instrument not suitable for on-site determinations, which demonstrate comparable LODs with the sensor developed for DON determination in wheat grain but shorter assay times, mainly due to the fact that the assay is executed in a single step. Imaging SPR (iSPR) has been also exploited for the multiplexed detection of mycotoxins, including DON, in cereals and related foods [38,39]. In the first of these two reports [38], the performance of a portable 6-plex instrument was compared to that of a 3-plex bench top instrument. Although the portable instrument had higher detection limits than the bench top one, its analytical performance was judged as more than satisfactory for on-site determinations. In the second report [39], gold nanoparticles were used as labels in a bench top iSPR instrument to enhance detection sensitivity of DON wheat. Both iSPR systems required matrix matched calibrators. Another optical sensing platform applied to DON detection in cereals was BLI [35,40,41]. The assay duration in all cases was shorter but the LODs were much higher than those achieved with the developed sensor. Finally, the WLRS assay developed is compared with an assay for DON determination in beer integrated onto silicon MZIs [42]. The LOD achieved with the MZIs was approximately three times lower than that obtained with the WLRS sensor for the same assay duration. In general, it can be stated that the WLRS assay developed for DON determination in wheat and maize whole grain is comparable both in terms of LOD and assay duration with the most relevant literature reports. The assay duration of the sensor developed could be shortened if the primary or the secondary or both antibodies were replaced by others of higher affinity or by using the antibodies already implemented after labelling to enhance the signal obtained by the label-free immunoreaction. It should be, however, noticed that the duration of tests performed with lateral flow devices range from 3 to 10 min, thus the duration of the developed assay is not significantly longer than that of the tests used almost exclusively at the point-of-need. In addition, the WLRS system holds the promise for easy adaptation to a portable low-cost instrument, while the proposed sample preparation method can be performed with low-cost and small-size instruments facilitating its application for on-site determinations. Regarding the instrument size, the current prototype has a footprint of 35 cm × 35 cm, which could be further decreased through a new design of certain modules such as the light source, the reflection probe and the module for handling the solutions. The cost of the chip depends heavily on the chip size and production volume. By considering a chip size reduction of 50%, which is easily feasible, and high-volume production, the cost of the chip for single use will be less than 1 euro. Furthermore, through the use of a single chip for 20 times (after regeneration), the cost of a single test will drop dramatically.

## Figures and Tables

**Table 1:** Mean percent recovery (%R) ± 1 standard deviation values determined for spiked maize samples after application of different extraction methods. Each sample was analyzed in triplicate.

Extraction Method	%R		
	20 ng/mL (1000 μg/kg)	50 ng/mL (2500 μg/kg)	200 ng/mL (10,000 μg/kg)
H_2_O	98.0 ± 4.0	97.0 ± 3.0	105 ± 4.0
MeOH/H_2_O 80:20	55.0 ± 4.5	57.0 ± 5.0	53.5 ± 4.0
MeOH/H_2_O 60:40	66.0 ± 3.0	64.0 ± 3.5	67.0 ± 4.0
MeOH/H_2_O 40:60	81.0 ± 4.0	78.0 ± 5.0	82.0 ± 3.0
MeOH/H_2_O 20:80	96.0 ± 2.0	94.0 ± 3.0	98.0 ± 3.0
AcN/H_2_O 80:20	92.0 ± 4.0	93.0 ± 2.5	95.0 ± 3.0
AcN/H_2_O 60:40	82.0 ± 3.0	85.0 ± 4.0	84.0 ± 3.5
AcN/H_2_O 40:60	85.0 ± 4.0	87.0 ± 2.5	83.0 ± 4.0
AcN/H_2_O 20:80	92.0 ± 2.0	89.0 ± 4.0	88.0 ± 3.0
H_2_O/OKM1	97.0 ± 3.5	98.0 ± 4.0	102 ± 5.0
H_2_O/AKM1	93.0 ± 3.0	96.0 ± 5.0	92.0 ± 3.0
H_2_O/ΕΝΚ1	100 ± 4.0	92.0 ± 3.0	95.0 ± 4.0

**Table 2:** Comparison of the White Light Reflectance Spectroscopy (WLRS) DON assay with other optical sensors reported in the literature.

Method	LOD (μg/kg)	Sample Type	Assay Duration (min)	EU Maximum Levels (μg/kg)	Ref. Number
WLRS	62.5	maize wheat	12(17 *)	1750	this work
SPR	50	wheat	5(7 *)	1750	36
SPR	57	wheat	4(6 *)	1750	37
	9	wheat-based breakfast cereal		500	
	6	maize-based baby food		200	
iSPR	64	barley	4(15 *)	1250	38
iSPR	15	wheat	<9(17.5 *)	1750	39
BLI	128	wheat	5(6.5 *)	1750	35
	737	wheat dust		-	
BLI	100	wheat flour	4(7 *)	750	40
BLI	90	wheat flour	4(6 *)	750	41
MZI	20	beer	12(17 *)	-	42

* Assay duration including regeneration step.
